# Supplementary material for: Impact of Foot Surgery and Pharmacological Treatments on Functionality and Pain in Rheumatoid Arthritis: A Five-Year Longitudinal Study
Source: Healthcare (Basel). 2025 Apr 27;13(9):1004. doi: 10.3390/healthcare13091004 (PMC12071480; doi:10.3390/healthcare13091004)
Supplement: Supplementary file 1 [file healthcare-13-01004-s001.zip › healthcare-3553820-supplementary.pdf]

Supplementary Table S1. Summary of Confounding and Pairwise Analyses

Outcome	Analysis Type	Method	Result
General Pain	Group comparison	Kruskal–Wallis	H = 4.89, $p = 0.299$
General Pain	Pairwise comparisons	Mann–Whitney U + Bonferroni	All $p > 0.005$
General Pain	Multivariable model	Linear regression	$p = 0.362$ (surgery effect)
General Pain	Adjustment	Propensity score (covariate)	$p = 0.362$
General Pain	Stratified analysis	DAS28 High/Low	$p = 0.270$ / 0.197
Foot Function Index	Group comparison	Kruskal–Wallis	H = 3.23, $p = 0.520$
Foot Function Index	Pairwise comparisons	Mann–Whitney U + Bonferroni	All $p > 0.05$
DAS28-PCR	Group comparison	Kruskal–Wallis	H = 6.22, $p = 0.184$

DAS28-PCR = Disease Activity Score using C-reactive protein.
